# Severe Pediatric Open Skull Fracture With Exposed Brain Matter: A Case Report

**DOI:** 10.7759/cureus.46521

**Published:** 2023-10-05

**Authors:** Sophia Mirkin, Michael Wu, Jessica Colon, Jose J Burgos, Fernando Perez

**Affiliations:** 1 Osteopathic Medicine, Dr. Kiran C. Patel College of Osteopathic Medicine, Nova Southeastern University, Fort Lauderdale, USA; 2 General Surgery, HCA Florida Kendall Hospital, Miami, USA; 3 Pediatric Critical Care, HCA Florida Kendall Hospital, Miami, USA

**Keywords:** pediatric trauma, traumatic brain injury, spontaneous decompressive craniotomy, intracranial pressure, open skull fracture

## Abstract

Head trauma in the pediatric population carries a high rate of morbidity and mortality. The major causes of head trauma are related to falls, recreational activities, motor vehicle accidents, and gunshot wounds. Traumatic brain injury (TBI) can occur after severe head trauma and is defined as an alteration in brain function, or other evidence of brain pathology, caused by an external force. Intracranial edema and herniation are common consequences of a TBI in pediatric patients and are commonly relieved via decompressive craniectomy.

This case study describes a 13-year-old male presenting to the trauma center after an unhelmeted all-terrain vehicle (ATV) accident with a positive head strike and loss of consciousness. The evaluation revealed extensive skull fractures extending from the frontal to the occipital lobe with brain exposure. Computed tomography (CT) scan of the head demonstrated extensive, open skull fractures with significant displacement of the exposed brain, extensive bilateral parietal and frontal bone fractures, and bilateral temporal bone displaced fractures more extensive on the left. A bilateral hemicraniectomy was performed due to diffuse cerebral edema and a left frontal ventriculostomy was placed to monitor and manage intracranial pressure (ICP).

It is believed that the unique presentation of an open skull fracture with an exposed brain acted as a decompressive method allowing for extreme lifesaving measures to be performed to save the patient. Further exploration is needed to truly understand the effects of the unique injury presentation and the role of an open fracture in the delay of increased ICP.

## Introduction

Head trauma in the pediatric population carries a high rate of morbidity and mortality as the skull has not reached its full maturity and offers less protection to the brain [1]. The skull is made up of the calvarium (frontal, parietal, temporal, and occipital bones) and the skull base (sphenoid, palatine, maxillary bones, and portions of the temporal and occipital bones). Skull fractures can be classified as linear, depressed, and open skull fractures [2]. Open skull fractures allow for communication between the skull and the outside of the scalp, exposing the brain to the environment outside of the closed skull. The major causes of head injuries in children are falls, recreational activities, motor vehicle accidents, and gunshot wounds. Motor vehicle accidents are most common in the four- to 17-year-old age group, and traumatic head injuries are more prevalent in boys than in girls [2].

Traumatic brain injury (TBI) can be defined as an alteration in brain function, or other evidence of brain pathology, caused by an external force [3]. Swelling and herniation of the brain are deadly secondary complications that can arise in the setting of a severe TBI [4]. Intracranial edema is another consequence of TBI that is highly dangerous as the rigid skull limits the space available for extra fluid to gather, leading to an increase in intracranial pressure (ICP) within the skull, subsequent herniation, and death [5]. Studies have found children are more likely to experience a rapid increase in diffuse brain edema after severe TBI than adults [6]. The medical and surgical aim is to prevent the early rise in ICP after a pediatric TBI as this has been linked to poorer outcomes [7]. 

Decompressive craniectomy has been a surgical method commonly employed in the treatment of pediatric TBI and increased ICP [8]. The method is aimed at decreasing the ICP in individuals resistant to medical treatment [8]. Although many different techniques have been described for decompressive craniectomy, universally, they include the removal of a portion of the skull to reduce swelling and pressure [9]. Various studies have been conducted to assess the efficacy of the surgical procedure. For instance, the RESCUEicp trial assessed 408 patients aged 10-65 years with TBI and increased ICP and found that usage of decompressive craniectomy would increase the number of survivors and percentage of individuals being independent at home after the procedure [10]. Signs of brain herniation and anisocoria are clear indications for the usage of decompressive craniectomy, and the procedure has shown promise as a treatment option for increased ICP after a pediatric TBI [10,11]. Temporal lobectomy may serve as an additional surgical intervention to help combat progressively increasing ICP after a severe TBI [12].

## Case presentation

A 13-year-old male with no past medical history presented to a Level 1 Trauma Center after an unhelmeted all-terrain vehicle (ATV) accident with a positive head strike with loss of consciousness. Bilateral chest needle decompression and intubation were performed before arrival. The primary survey revealed bilateral and equal breath sounds, symmetrical expansion, regular rate and rhythm, normotensive, pulses in all extremities, Glasgow Coma Scale (GCS) of 3, and dilated and fixed pupils. The evaluation revealed an extensive skull fracture from the frontal to occipital lobe and exposure of the brain with no other obvious deformities, lacerations, or ecchymosis. 

**Figure 1:**
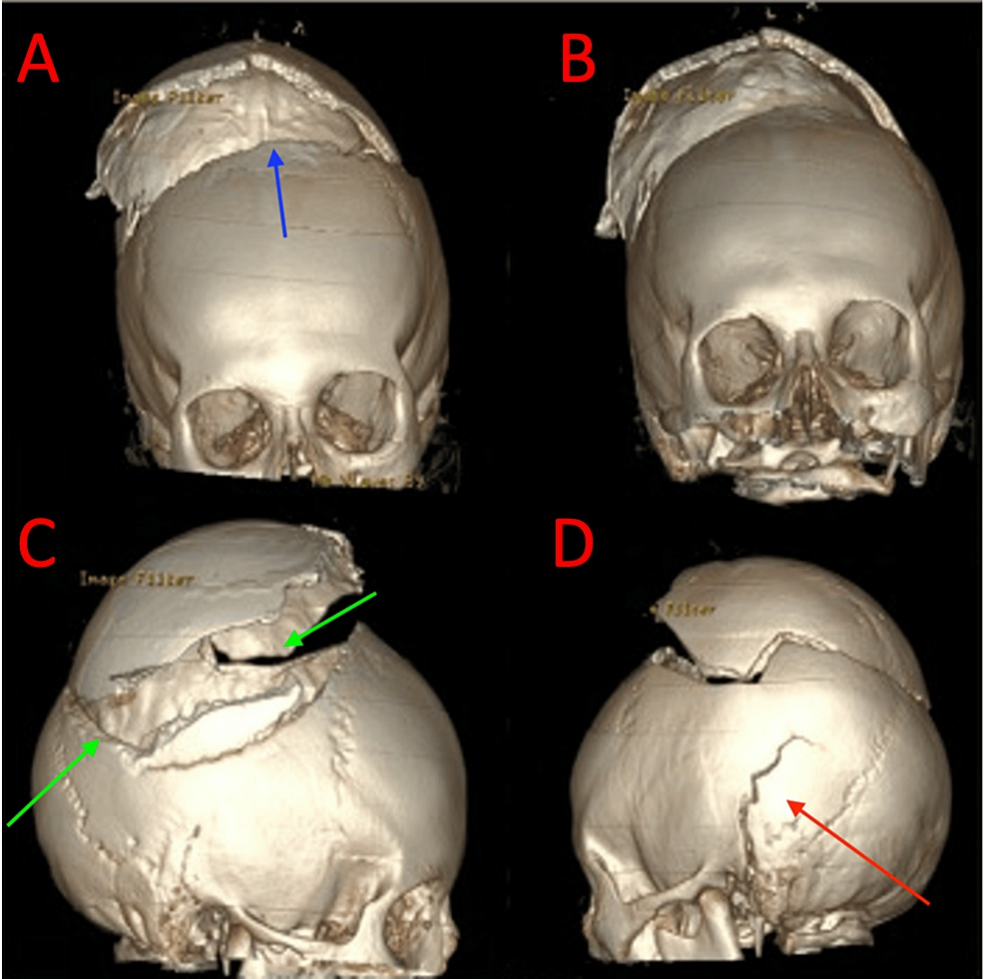
Extensive open skull fractures. Computed tomography volume-rendered three-dimensional (3-D) reformats demonstrating comminuted, displaced, open calvarial fractures involving the bilateral temporal (red arrow) and parietal bones (green arrows), as well as the frontal bone (blue arrow).

**Figure 2:**
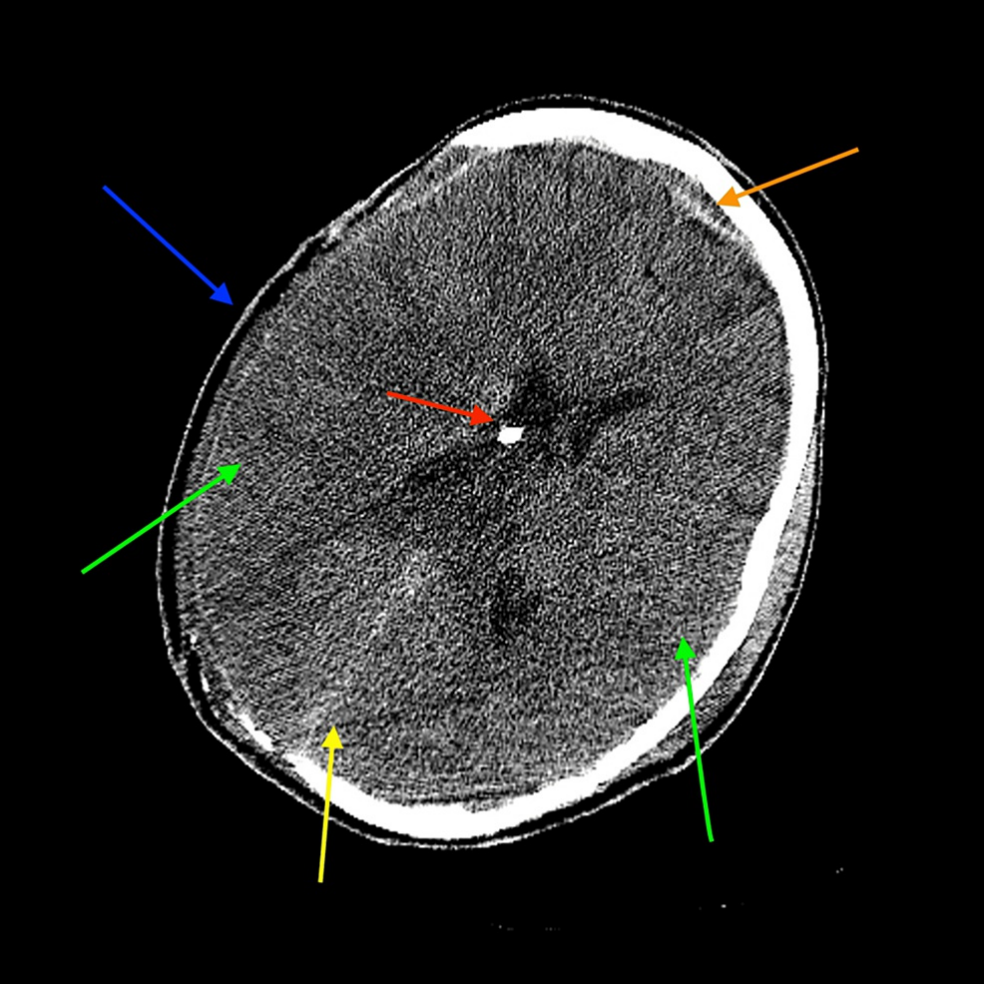
Axial reformat non-contrast CT of the brain at the level of the lateral ventricles. Axial reformat non-contrast CT of the brain at the level of the lateral ventricles demonstrating a right-sided open parietal calvarial defect (blue arrow), diffuse reactive cerebral edema with sulcal effacement (green arrows), right-to-left midline shift, and a partially visualized ventriculostomy catheter in the body of the right lateral ventricle (red arrow). Additionally, there are frontal epidural (orange arrow) and parafalcine blood products (yellow arrow). CT, computed tomography

**Figure 3:**
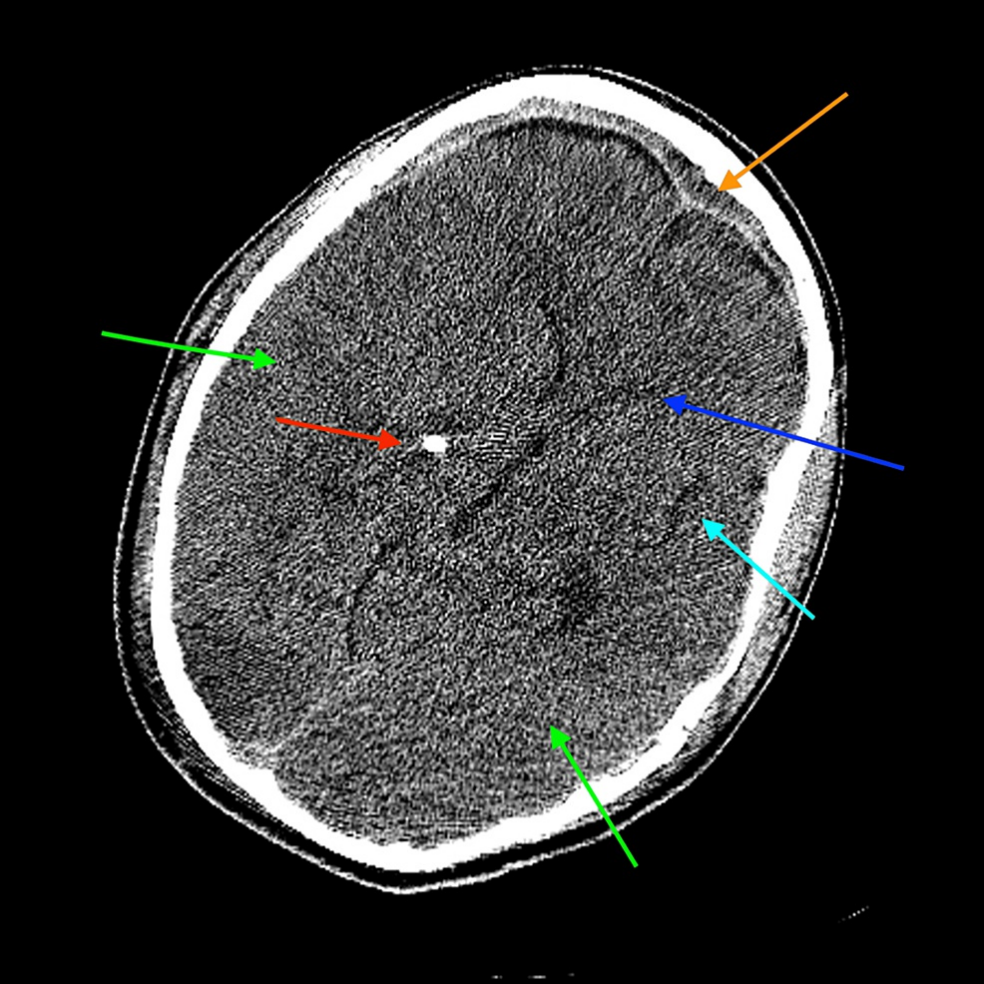
Axial reformat non-contrast CT of the brain at the level of the basal ganglia. Axial reformat non-contrast CT of the brain at the level of the basal ganglia demonstrating frontal and right parietal epidural blood products (orange arrow), diffuse reactive cerebral edema with resultant mass effect, and effacement of the lateral ventricles (dark blue arrow), basal cistern, and sulci (light blue arrow), as well a loss of the gray-white matter differentiation (green arrows). A partially visualized ventriculostomy catheter is present adjacent to the third ventricle (red arrow). CT, computed tomography

Computed tomography (CT) scan of the brain without contrast revealed extensive, open skull fractures with significant displacement of the exposed brain, extensive bilateral parietal and frontal bone fractures, and bilateral temporal bone displaced fractures more extensive on the left (Figures 1A-1D). Additionally, an 8-mm frontal epidural hematoma with displacement of the superior sagittal sinus was noted (Figure 2). Loss of normal gray-white matter differentiation, along with brain edema and significant effacement of the ventricular system and basal cisterns, was observed (Figure 3). CT venogram did not demonstrate any disruption of the superior sagittal sinus. No spinal stenosis, extravasation, or facial bone fractures were reported.

Aggressive lifesaving interventions were pursued after discussion with the family. The patient was diagnosed with severe TBI, open right frontoparietal skull fracture, right scalp laceration, diffuse cerebral edema, and obstructive hydrocephalus. Surgical intervention was deemed necessary to repair the open frontoparietal skull laceration, and bilateral decompressive hemicraniectomy was performed due to the diffuse cerebral edema. Placement of left frontal ventriculostomy was also performed to monitor and manage possible elevation in ICP. Both hemicraniectomy and ventriculostomy were completed without complication. 

The patient was started on antibiotics for open bone fracture and meningitis prophylaxis. TBI-induced syndrome of inappropriate antidiuretic hormone (SIADH) developed with subsequent diabetes insipidus. A neurology consult revealed a new GCS score of 5. After consultation with neurosurgery, Keppra 500 mg/5 mL was initiated to prevent seizures, and electrolytes were replaced with blood pressure parameters of systolic blood pressure less than 140 mmHg and mean arterial pressure between 70 and 90 mmHg, head of bed elevation to 45°, and ICP goal of less than 20 mmHg. Right and left chest tubes were removed on postoperative days 4 (right) and 5 (left).

On postoperative days 9 and 10, completion scans were performed. CT scan and magnetic resonance imaging (MRI) of the brain revealed no additional findings. Thirteen days post-admission, after extensive conversation with the family, it was decided to transfer the patient to a nearby children’s hospital for further treatment.

## Discussion

**Figure 4:**
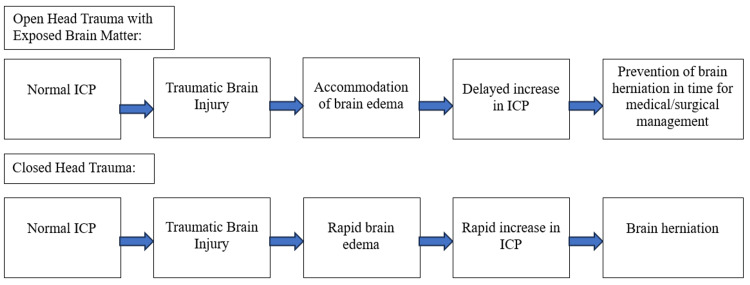
Flowchart depicting the sequence of events of an open and closed head injury after a pediatric TBI. The proposed model suggests that the open head injury allowed for the accommodation of brain edema, delay in increased intracranial pressure, and subsequent prevention of brain herniation in time for medical/surgical management. Data from [13]. ICP, intracranial pressure; TBI, traumatic brain injury

We present a case of a 13-year-old male with an extensive open skull fracture due to an unhelmeted ATV accident. After extensive medical and surgical intervention, the patient was able to be stabilized and transferred to a nearby children’s hospital for better-suited care. It is believed that the unique presentation of the open skull fracture with significant displacement of the exposed brain may have been a contributing factor to the patient's survival and allowed for subsequent stabilization and transfer (Figure 4). 

With the radical nature of the injury in the presented case, it is believed that the exposure of the brain matter from the open skull fractures could have served as a method to combat brain swelling and herniation, leading to the avoidance of early death. Vankipuram et al. presented a case of an eight-day-old neonate whose parietal and temporal bone fractures acted as a spontaneous decompressive craniotomy, allowing the brain to swell without the constraints of a rigid skull [14]. Similarly, our patient’s extensive bilateral parietal, frontal, and temporal bone fractures could have served as additional space for the accommodation of brain edema and potentially been the leading factor for a delayed increase in ICP and prevention of herniation in the acute setting. The literature has shown the timing of decompressive craniectomy is linked to a more favorable prognosis in the pediatric population. For instance, Taylor et al. found very early decompressive craniectomy in children with TBIs obtained a greater reduction in ICP, fewer episodes of intracranial hypertension, and a better quality of life than when using medical management alone (54% vs. 14%) [15]. However, there is limited research on the potential role of spontaneous decompressive craniectomy in reducing ICP after an open skull fracture in a severe pediatric TBI. Furthermore, Ren et al. aimed to investigate the influence of skull fractures on TBI induced by blunt trauma and found that a skull fracture could reduce the risk of diffuse brain injury significantly under medium and high velocities [16]. It is believed that the reduced risk of diffuse brain injury is largely due to the absorption of energy from the skull fracture [17]. The impact energy absorbed by the skull fracture is thought to reduce the energy transferred to the brain tissue and serve as a protective mechanism for the brain [16]. More research needs to focus on evaluating the effect of pediatric open skull fractures specifically and their role in potentially shielding the brain from additional trauma. After craniectomy and recovery, the acrylic prosthesis may be performed to protect the brain and prevent sinking skin flap syndrome [18].

TBIs are commonly classified using GCS, which assigns patients a numerical value to assess various cognitive and motor functions. In a review conducted by Goldberg et al., a low GCS has been shown to correlate with poorer outcomes and higher mortality rates while a decreasing GCS is more predictive of a poorer outcome than an initially low GCS [19]. Comparatively, an uptrending GCS favors improved outcomes and survival rates. Our patient initially presented to the trauma center with a GCS of 3 and later improved to a GCS of 5 after extensive surgical and medical intervention. Although still classified as severe, the uptrend in the score is believed to reflect a more favorable outcome, which is suspected to be a factor leading to the stabilization of the patient while hospitalized.

In our review of the scientific literature, we found no similar reported cases of a pediatric spontaneous decompressive craniotomy from an extensive, open skull fracture with exposed brain matter. Other case reports most applicable to pediatric TBIs focus on neonates with open sutures, surgical and medical management, or limited resources in a third-world country. To date, the finite studies available do not examine the effects of a spontaneous decompression craniotomy after a trauma-induced open skull fracture in an adolescent. Consequently, a major limitation of our study is the sparsity of research available regarding pediatric open skull fractures and their effect on the brain. We hope our report brings to light the limited literature on this topic and encourages further research in pediatric TBIs.

## Conclusions

Head trauma in the pediatric population carries a high rate of morbidity and mortality. The unique presentation of the open skull fracture with significant displacement of the exposed brain is believed to have been a factor leading to the patient's survival. Spontaneous decompressive craniotomy from open skull fractures has not been extensively researched and needs to be further explored as a potential factor in preventing early death in a pediatric TBI. This case highlights the potential role spontaneous decompressive craniotomy has in allowing for the accommodation of brain swelling and delay of increased ICP, risk of herniation, and subsequent death.
